# Effects of Loss of Consciousness upon Memory of Preceding Events

**Published:** 1876-08

**Authors:** 


### Effect of the Loss of Consciousness upon the Memory of Preceding Events.

In the *Sanitarian*, February, 1876, Prof. Frank H. Hamilton, M. D., President of the Medico-Legal Society, New York, etc., has the following valuable remarks on this topic:

Some years since, I was consulted by a lawyer of considerable experience in criminal jurisprudence, in relation to the case of a client who had been arrested for assault, under the following circumstances: A woman occupying an apartment alone in a tenement house, charged that she had been assaulted in the night, while in bed, by a man wearing a mask; that during the struggle which ensued the man struck her with a club upon her head, rendering her insensible; and that she remained in this condition till morning, when, consciousness having returned, she called in some one who was passing. She was found with her clothes on, lying upon the ouside of the bed, and with a wound, accompanied by considerable swelling, upon her forehead. She then stated how she had been assaulted, declaring that she recognised the man, and upon her statement my friend's client was soon arrested.

I gave it as my opinion that, if she was insensible during several hours following the receipt of the injury, her testimony as to what transpired immediately preceding the unconsciousness ought not to be allowed much weight; and that the fact that she claimed to be able to recall all the events, up to the final blow so distinctly, furnished, to say the least, some ground for suspicion that it was all a fraud.

Subsequently, it was ascertained that this woman, who had borne a doubtful reputation among her neighbors, had been found drunk in the streets on the night of the assumed assault, and that another woman had lifted her up and conducted her as far as the door of her house. The case was dropped; but my attention being called by the value of testimony given under such circumstances, and from that time until the present I have continued to make and record occasional observations upon the subject, as opportunities have been afforded. My observations, have, indeed, been quite numerous, but only those which have been carefully recorded constitute the basis of my present remarks.

South, in his notes to Chelius' Surgery, mentions a case in which a woman in a comatose condition, suspected that it was due to extravasation of the blood, and applied the trephine, when, upon the escape of imprisoned blood, the woman recovered her senses promptly, "and was able to give a clear account of the manner in which the accident occurred." No doubt the writer reported the facts in this case correctly, and there was no failure in the memory of preceding events, but this is not what always happens; indeed, in a case of my own, of a very similar nature, the patient having been blown up in a steamboat explosion, causing a fracture of the skull and a rupture of the middle meningeal artery, incision of dura mater brought relief and restored consciousness; but although his recovery was complete, he was never able to recall any incident connected with the disaster, or any event of the day on which the accident occurred.

My experience has also led me to regard as appochrypal those stories which are now and then related in daily newspapers, and sometimes in journals devoted to science, of persons, who, having been stricken down with sudden loss of consciousness while speaking, have, upon the restoration of consciousness after the lapse of many hours or days, proceeded to finish the broken sentence, or have taken up their thread of thought as if no interruption had occurred. When the conscience has been transient or momentary, I think I have seen this happen; but not when it has been complete and prolonged. It may not be proper to deny positively that such a case has ever occurred; but no such example has ever come under my observation, nor have I seen recorded a case properly authenticated.

It is apparent that the questions which this subject opens, as to the admissibility of the patient's testimony under these conditions, have a very wide application, and that, if possible they ought to be determined by a reference to carefully observed facts.

Complete but temporary unconsciousness may be occasioned by a blow upon the head, causing concussion of the brain; by compression of the brain, ensuing from a fracture of the skull with depression of the fragments; by extravasation of blood within the skull; by embolism of the cerebral artery; by syncope; by epilepsy; by the inhalation of anæsthetics; by alcoholic inebriation, which is only a form of anæsthesia; by the use of opiates and other narcotics; and, perhaps, by many other causes.

Even sleep, a natural event, which occurs without any positive cerebral disturbance, may possibly, at times, cause a break or a failure in the memory. At all events, it is a proper question for consideration.

In the large majority of cases, however, which come under the observation of the medical jurist, the insensibility will be found to be due to concussion and compression of the brain, caused by blows inflicted upon the head, or by the shock incident to railroad or other serious accidents; and it is quite probable that the laws which govern the recollection of preceding events will be found, upon thorough examination, to vary according as the loss of consciousness may be due to one or the other of the many causes above enumerated; but my observations are not at present sufficiently extensive to enable me to note such differences, if they actually exist. No attempt will be made, therefore, to do more than lay before you a few facts, and to suggest a few general inferences, but with no pretence that these inferences are entitled to be considered in the character of a well-established law.

You will better appreciate the pertinence of the examples if we reverse the usual order of consideration, and place before you briefly the conclusions, before reporting the cases from which the conclusions have been mainly drawn.

1. A temporary loss of consciousness does, in many cases, impair the recollection of events immediately preceding.

2. In some cases it does not.

3. Impairment of memory, caused by loss of consciousness, is of two kinds:

(*a*) Complete obliteration of memory, dating from a period of time or from an event which the patient can distinctly name.

(*b*) Events may be remembered up to the moment of the receipt of the injury, but with a degree of confusion and uncertainty.

4. In the case of complete and sudden obliteration of memory, dating from a period preceding the injury, the length of this period will, in general, have relation to the degree and length of the insensibility. If the patient is unconscious but a few moments, of if the loss of consciousness is only partial, he may recall events nearly or quite up to the moment of the receipt of the injury. If, on the other hand, the loss of consciousness is complete, coming to coma, and prolonged, it is probable that he will have forgotten events for some time preceding the injury.

In reference to that class of cases in which the memory of events is retained up to the the moment of the accession of unconsciousness, but in which the memory is confused and uncertain, it may be observed that they are likely to possess the most importance in a medico-legal point of view. Those who have forgotten completely preceeding events will make no statements in relation to these events unless they fully intend to swear falsely and to practice a fraud, and we may hope that the number of persons who will thus willfully perjure themselves is not large; but the other class of persons may testify honestly, and yet very erroneously, their memory of preceding events being in part correct and part false. There may be just enough truth in their statements to give a color, and to induce the court to accept of the whole without qualification or abatement. Under these circumstances the witness may, unintentionally, put language in the mouth of one which was uttered by another. He may transpose the order of events, or in many other ways deviate from the exact truth, without knowing that he does so. He may even mistake the visions which flit through his mind, while unconscious, for facts; or, what is more likely to happen, he may receive from the conversation of those who are about him, when consciousness is returning, impressions as to what happened before the accident, which will assume the character of convictions, and will be eventually regarded by him as having been recalled by an act of his own memory.

Recently a case has come under my notice which may illustrate the danger of accepting testimony of the class last described.

In the case of Filer against the New York Central R. R. Co., Supreme Court, Monroe County, N. Y., January Circuit for 1873, it was in evidence that Mrs. Filer, in leaving the cars while under motion, at Fort Plain, was caught by her dress, thrown down, and dragged some distance; from which it was claimed she suffered severe and permanent bodily injury. She was conscious after the accident for at least half an hour, and perhaps longer.

Being brought to the stand, Mrs. Filer testified she was instructed by the brakeman to leave the car while it was in motion, thus throwing the responsibility of the act upon the Company; that when she fell, she "gave one terrible scream;" that a gentleman, whom she particularly designated, shouted, "She is killed." * * "Then the bell rang," and from this moment she lost all consciousness until she found herself seated in a chair in the sitting room of the depot.

Her statement that the breakman told her to step down, as the cars would not stop any more, is contradicted by the brakeman himself; and there are several other points in her testimony, relating to the manner of the accident, which are denied.

The case was tried again, on appeal, in July, 1875. In this last trial I was called to the stand as an expert, particularly in reference to the character of the injuries she received; but incidentally I gave it as my opiuion that Mrs. Filer's testimony, ought, under the circumstances, to be received with great caution, although I entertained no doubt but that she testified to what she thought was true.

The jury gave a verdict again for the plaintiff, with increased damages.

In this case there was no evidence that the loss of conscionsness was caused by concussion of the brain, or any other bodily injury; but it appeared to be caused by fright alone. I have no reason, however, to suppose that this fact effects the principle involved, or the rule, if any, exists, governing the memory of preceding events, except as it renders it more probable that the unconsciousness was transient and slight; but the evidence went to show that it was not very transient or incomplete.

It will be observed that, in the brief presentation I have made of this subject, I have done little more than to open the ground for discussion and for future study. Some experience in examining these cases renders it proper, however, for me to suggest to those who undertake to prosecute similar investigations, that they will have to exercise great care in their examinations, rejecting, of course, all who were intoxicated before the receipt of the injury, or who were not sufficiently intelligent or honest to be trusted in their statements, or had not completely recovered their intelligence since the receipt of the injury. The inquisition should be close and critical, even with those who intend to speak the truth, since it will often happen that persons of this class will declare at first that they can recall distinctly the last circumstance preceding the unconsciousness, when in fact they cannot.—*Half-Yearly Compendium.*

